# Formation of α- and β-Cembratriene-Diols in Tobacco (*Nicotiana tabacum* L.) Is Regulated by Jasmonate-Signaling Components via Manipulating Multiple Cembranoid Synthetic Genes

**DOI:** 10.3390/molecules23102511

**Published:** 2018-09-30

**Authors:** Jinkai Sui, Chunkai Wang, Xiaofeng Liu, Ning Fang, Yanhua Liu, Wenjing Wang, Ning Yan, Huai-Bao Zhang, Yongmei Du, Xinmin Liu, Tiegang Lu, Zhongfeng Zhang, Hongbo Zhang

**Affiliations:** 1 Tobacco Research Institute, Chinese Academy of Agricultural Sciences, Qingdao 266101, China; suijinkai@163.com (J.S.); wangchunkai1990@outlook.com (C.W.); liuxiaofeng626@126.com (X.L.); fangning010@163.com (N.F.); liuyanhua@caas.cn (Y.L.); wangwenjing@caas.cn (W.W.); yanning@caas.cn (N.Y.); zhanghuaibao@caas.cn (H.-B.Z.); duyongmei@caas.cn (Y.D.); liuxinmin@caas.cn (X.L.); zhangzhongfeng@caas.cn (Z.Z.); 2 Biotechnology Research Institute, Chinese Academy of Agricultural Sciences, Beijing 100081, China; lutiegang@caas.cn

**Keywords:** tobacco, cembratriene-diol, secondary metabolism, jasmonate, COI1

## Abstract

Cembranoids are a group of natural diterpenoid compounds with pharmaceutical potentials, and the cembratriene-diols produced by *Nicotiana* (tobacco) species display activities in anti-nicotine addiction and neuron protection. Although the enzymes catalyzing cembratriene-diols’ formation in tobacco have been investigated, the regulatory mechanism underlying this physiological process remains unknown. This study has investigated the roles of phytohormone jasmonic acid (JA) in regulating cembratriene-diol formation in *N. tabacum* cv. TN90 and found that JA and COI1, the receptor protein of the bioactive derivative of JA (i.e., JA-Ile), display critical roles in regulating cembratriene-diols’ formation and the expression of cembranoid synthetic genes *CBTS*, *P450* and *NtLTP1*. Further studies showed that over-expressing either the gene encoding bHLH transcription factor *MYC2a* or that encoding MYB transcription factor *MYB305* could upregulate the cembranoid synthetic genes and enhance the cembranoid production in plants with dysfunction of COI1. Further studies suggest that COI1 and its downstream regulators MYC2a and MYB305 also modulate the trichome secretion, which is correlated with cembranoid formation. Taken together, this study has demonstrated a critical role of JA-signaling components in governing the cembratriene-diol formation and the transcription of cembratriene-diol synthetic genes in tobacco. Findings in this study are of great importance to reveal the molecular regulatory mechanism underlying cembranoid synthesis.

## 1. Introduction

Cembranoids, a group of natural diterpenoid compounds structurally composed of a 14-carbon cembrane ring with featured oxygen-containing substitutes, are widespread in nature, and currently, hundreds of cembranoids have been reported from plants (conifers and tobacco), insects, alligators and marine organisms [1]. In recent years, the biological activities of cembranoids in antimicrobial, anti-cancer and anti-inflammation effects, as well as its fascinating architectures, have attracted great interest from researchers of natural products and pharmaceuticals [1,2]. *Nicotiana* (tobacco) species are the terrestrial plants most abundant in cembranoids, which contribute to the characteristic aroma of tobacco. The two major cembranoids in tobacco are *4S*-cembranoid (*1S*,*2E*,*4S*,*6R*,*7E*,*11E*)-cembra-2,7,11-triene-4,6-diol (α-cembratriene-diol) and its *4R* epimer (β-cembratriene-diol) [1]. Intriguingly, the tobacco β-cembratriene-diol also displays neuroprotective and nicotine anti-addictive activities via binding to the nicotinic acetylcholine receptors (nAChR’s) [1], implying that tobacco plants produce both addictive (alkaloids) and anti-addictive compounds.

**Figure 1:**
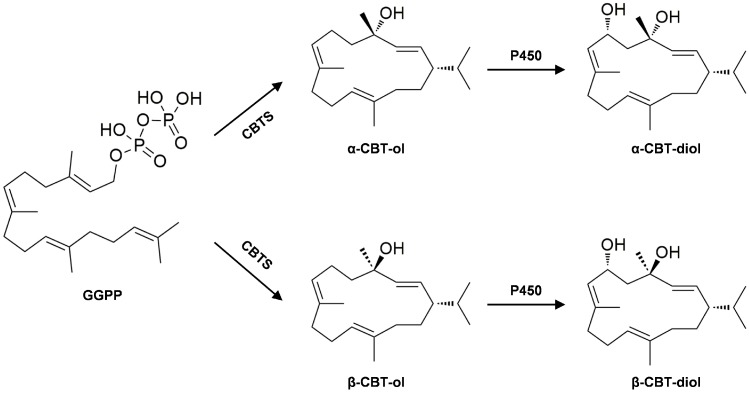
The biosynthetic pathways of the cembratriene-diols in tobacco [1]. GGPP, geranylgeranyl diphosphate; CBT, cembratriene.

Cembranoids are synthesized in the glandular trichome, known as phytochemical factories, producing a large portion of plant metabolic compounds, such as polysaccharides, terpenes, phenylpropanoids and flavonoids [3,4,5]. Previously, the formation of cembranoids was investigated by multiple studies. The first step of cembranoid biosynthesis is catalyzed by cembratriene-ol synthase (CBTS) (Figure 1), which produces α- and β-cembratriene-ols from geranylgeranyl diphosphate (GGPP) [1]. The amount of cembratriene-ol production was increased by transient expressing of *CBT2a* [6], while the production of cembratriene-ol and cembratriene-diol was reduced by *CBTS* (*NtCYC1*) silencing in tobacco [7]. The second step of cembranoid biosynthesis is catalyzed by cytochrome P450 hydroxylase (P450) (Figure 1), which catalyzes the hydroxylation of cembratriene-ols to form cembratriene-diols [8]. Suppression of *P450* caused a decrease in the content of cembratriene-diols, but an increase in the content of cembratriene-ols in tobacco [8]. Moreover, tobacco lipid transfer proteins (NtLTP1) play important roles in lipid secretion from glandular trichomes [9]. Over-expressing *NtLTP1* in tobacco resulted in the increased secretion from glandular trichomes, while silencing of this gene led to reduced trichome secretion [9]. Although enzymes catalyzing multiple steps of cembranoid formation in tobacco have been identified by a number of studies [10,11], the regulatory mechanism underlying this physiological process remains largely unknown.

Since glandular trichomes function as the factory producing cembranoids, this implies a critical role of trichome-developmental regulators in governing cembranoid synthesis. It was shown that the initiation, development and secretion of plant trichomes were regulated by several phytohormones, including jasmonate acid (JA), 6-benzylaminopurine (BAP) and gibberellin (GA) [12,13,14,15,16,17]. Studies in *Artemisia annua* indicated that JA and BAP positively regulate the density of glandular trichomes, but only JA could promote the secretion of glandular trichomes [16], suggesting a pivotal role of JA in regulating trichome secretion. JAs are a kind of fatty-acid-derived hormone functioning in plant responses to wounding and herbivore attacks and play important roles in regulating plant development, defense responses, secondary metabolism and other physiological processes [18,19,20,21]. In *Arabidopsis*, the initiation and formation of trichomes can be greatly increased by herbivore attacks and JA [17,22], and proper JA perception is crucial for trichome induction [23]. Studies on Solanaceae plants showed that dysfunction of COI1 could suppress the development of glandular trichome in tobacco and notably reduce the secretion of glandular trichome in tobacco and tomato [24,25]. These facts suggested that JA, as well as COI1, the receptor protein of the bioactive derivative of JA (i.e., JA-Ile), display crucial roles in the development and secreting of trichomes.

Previous studies have established the molecular model of JA-signaling in plants. Jasmonoyl-isoleucine (JA-Ile), the bioactive derivative of JA, is perceived and transduced by the SCF^COI1^(Skip/Cullin/F-box) complex [20,26], which results in the ubiquitination and subsequent degradation of JAZ (jasmonate ZIM domain) proteins, the repressors of JA-signaling, via the 26S proteasome pathway [27,28]. Then, the downstream bHLH (such as MYC2) and MYB transcription factors are released from the JAZ repressors to activate JA-signaling responses [27,29,30]. Studies in a number of plants have shown that the bHLH transcription factor MYC2 acts as a master regulator mediating multiple aspects of JA responses [31,32,33]. The R2R3 MYB transcription factor MYB305 from *Antirrhinum* was demonstrated to regulate the biosynthesis of flavonoid in flowers, and its homologue NtMYB305 from tobacco was shown to modulate the carbohydrate metabolism in both vegetative and reproductive organs [25,34,35]. Previously, cumulative studies in multiple plants have suggested that the WD40-bHLH-MYB complex displays a critical role in modulating the downstream JA responses, including trichome development, secondary metabolism and transcriptional regulations [14,15,36,37,38,39], and a group of transcription factors are involved in this regulatory network [10].

Even though the roles of JA in regulating plant secondary metabolism have been intensively investigate, its function in cembranoid synthesis remain largely unknown. This study has analyzed the induction of cembranoid synthesis by JA in tobacco (*N. tabacum* cv. TN90) and investigated the mechanism of JA-signaling components including COI1, MYC2 and MYB305 in governing the cembranoid formation and modulating the transcription of cembranoid synthetic genes in tobacco. Findings of this study have provided important information for dissecting the molecular regulation of cembranoid synthesis in tobacco.

## 2. Results

### 2.1. The Formation of Cembratriene-Diol Is JA-Inducible in Tobacco

**Figure 2:**
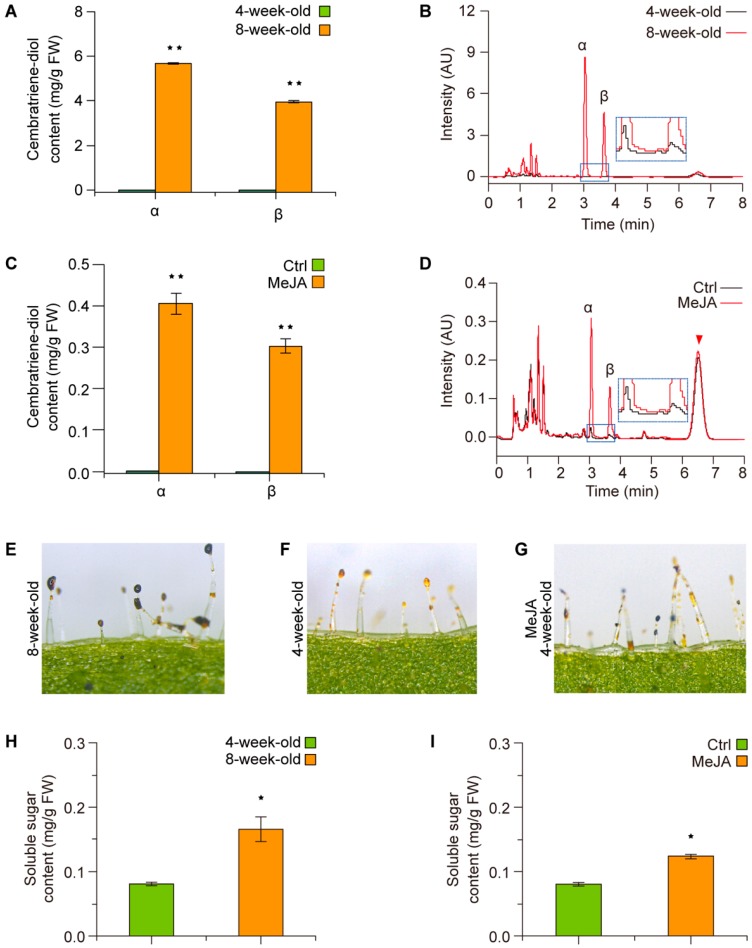
JA treatment improved the production of cembratriene-diol in tobacco. (**A**) Comparison of cembratriene-diol content between the four-week-old and eight-week-old tobacco plants. (**B**) The representative chromatogram profiles of cembratriene-diol determination by the UPLC system for the four-week-old and eight-week-old plants. (**C**) Determination of cembratriene-diol content between the control (Ctrl) and MeJA-treated four-week-old tobacco plants. (**D**) The representative chromatogram profiles of cembratriene-diol determination by the UPLC system for the control and MeJA-treated four-week-old plants. The triangle indicates an unidentified compound. (**E**–**G**) Trichome secretion droplets stained by I_2_/KI. (**H**,**I**) Soluble sugar content of the trichome secretion droplets. Values in (**A**,**C**,**H**,**I**) are the average of the data from three plants with triplicate measurements. Asterisks in (**A**,**C**,**H**,**I**) indicate significant difference from the four-week-old plants or the control treatment. Insets in (**B**,**D**) show the enlargements of the chromatogram images in the blue rectangles, and α and β in the graphs of (**B**,**D**) indicate α-cembratriene-diol and β-cembratriene-diol, respectively. Error bars, mean ± SE.

To investigate the roles of JA in regulating cembranoid synthesis, the four-week-old tobacco seedlings were selected to determine the induction of cembratriene-diol formation by JA, for the seedlings at this stage had mid-sized leaves with much lower cembratriene-diol content than the aged plants (Figure 2A,B, showing a comparison with the eight-week-old plants) and were suitable for the observation of JA-induction effects. The results showed that MeJA-treatment for seven days could increase the α and β-cembratriene-diol to approximately 0.4 and 0.3 mg/g FW (fresh weight) from a trace amount level of the control (Figure 2C,D).

Since cembranoids were secreted by the glandular trichomes on tobacco, the trichome secretion were observed under stereo-microscope after staining with I_2_/KI solution [1% (*w*/*v*) I_2_ in 3% (*w*/*v*) KI (potassium iodide)]. The observations showed that the color of the trichome secretion droplets of the young plants was much lighter than those of the aged (eight-week-old) plants (Figure 2E,F). While, the trichome secretion droplets of the MeJA-treated plants turned to a much darker color compared with that of the control, their color was still lighter than those of the eight-week-old plants (Figure 2G). Further analysis indicated that the trichome secretion droplets of eight-week-old plants contained a higher soluble sugar content than those of the four-week-old plants; moreover, MeJA treatment could increase the soluble sugar content of the trichome secretions (Figure 2H,I). These facts suggested that the cembratriene-diol formation in tobacco is regulated by JA, which may affect the composition of glandular trichome secretion. 

### 2.2. JA Displays a Role in Regulating the Transcription of Cembratriene-Diol Synthetic Genes in Tobacco

**Figure 3:**
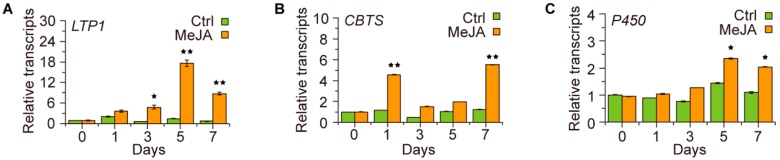
Effects of JA-treatment on the expression level of *LTP1*, *CBTS* and *P450* genes. (**A**–**C**) MeJA-induced transcription profiles of the cembranoid synthetic genes *LTP1*, *CBTS* and *P450*. Ctrl, plants of control treatment with H_2_O; MeJA, plants of MeJA treatment for indicated time points. The transcription level of each gene in the plants of control treatment at the “0” time point was arbitrarily set as “1”; values in (**A**–**C**) are the average of data from three plants with triplicate measurements. Asterisks indicate significant difference to the control treatment of the same time point in (**A**–**C**). Error bars, mean ± SE.

To figure out the molecular mechanism underlying JA-regulated cembratriene-diol formation in tobacco, we carried out a transcriptional assay to determine whether the cembratriene-diol synthetic genes are regulated by JA treatment. The transcription levels of a set of cembratriene-diol synthetic genes, including *LTP1* (lipid transfer protein), *CBTS* (cembratriene-ol synthase) and *P450* (cytochrome P450 hydroxylase) [10,11], were analyzed in the qRT-PCR assays. The results showed that the expression level of *LTP1* was increased to five folds of that in the control after three days of JA-treatment and to 17 folds of that in the control after five days of treatment (Figure 3A); *CBTS* was upregulated by four-fold after seven days of JA treatment (Figure 3B); and *P450* was increased by two-fold after five days of treatment (Figure 3C). These findings suggest that JA functions as a regulator of the cembratriene-diol synthetic genes.

### 2.3. COI1 Is Required for the Formation of Cembratriene-Diols in Tobacco 

**Figure 4:**
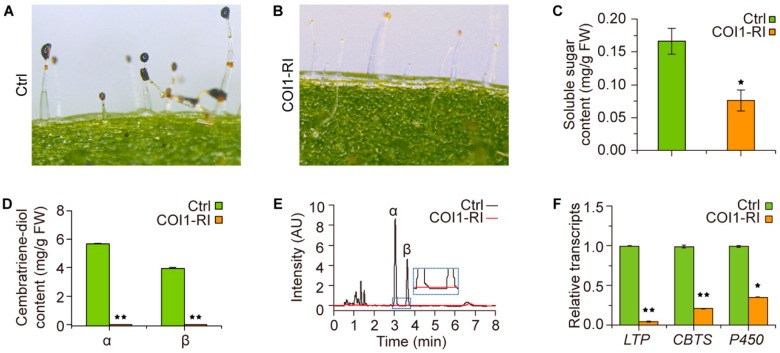
COI1 is required for the synthesis of tobacco cembratriene-diols. (**A**,**B**) Trichome secretion droplets stained by I_2_/KI. (**C**) Soluble sugar content of the trichome secretion droplets. (**D**) Comparison of cembratriene-diol contents between the control (Ctrl) and plants with dysfunction of COI1 (COI1-RI). (**E**) The representative chromatogram profiles of cembratriene-diol determination by the UPLC system for the control and plants with dysfunction of COI1. (**F**) Transcription profiles of the cembranoid synthetic genes *LTP1*, *CBTS* and *P450* in the control and plants with dysfunction of COI1. The transcription level of each gene in the control plants was arbitrarily set as “1”. Values in (**C**,**D**,**F**) are the average of data from three independent lines with triplicate measurements. Asterisks in (**C**,**D**,**F**) indicate significant difference from the control plants. The inset in (**E**) shows the enlargement of the chromatogram image in the blue rectangle, and α and β in the graph of (**E**) indicate α-cembratriene-diol and β-cembratriene-diol, respectively. Error bars, mean ± SE.

In order to uncover the mechanism of the JA-signaling pathway in regulating cembratriene-diol formation, tobacco plants with dysfunction of COI1 [25] were adopted for further analyses. Our previous studies showed that the trichome secretion of these plants was decreased to an extremely lower level (Figure 4A,B) [25]. The cembratriene-diol determination with the eight-week-old plants showed that the α- and β-cembratriene-diol contents of plants with dysfunction of COI1 were decreased to an undetectable level, while those of the control plants were 5.68 and 3.96 mg/g FW, respectively (Figure 4D,E). The transcriptional analyses revealed that dysfunction of COI1 greatly attenuated the expression *LTP1*, *CBTS* and *P450* genes (Figure 4F). Additionally, the plants with dysfunction of COI1 possessed significantly decreased soluble sugar content of the trichome secretions (Figure 4C). These results further demonstrated the importance of proper JA-signaling for cembratriene-diol synthesis in tobacco. 

### 2.4. Regulatory Roles of NtMYC2a in COI1-Mediated Cembratriene-Diol Formation in Tobacco

**Figure 5:**
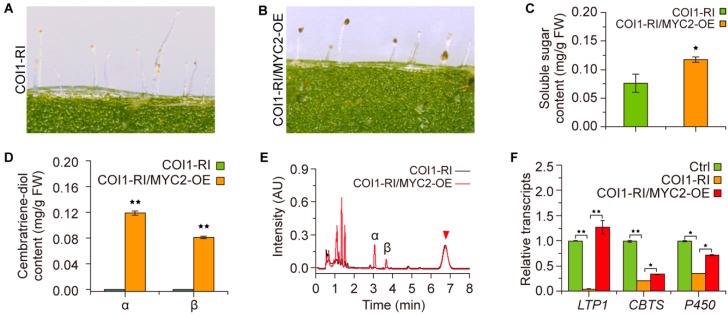
Regulatory roles of NtMYC2a in COI1-mediated cembratriene-diol formation in tobacco. (**A**,**B**) Trichome secretion droplets staining with I_2_/KI for plants with dysfunction of COI1 (COI1-RI) and overexpressing *NtMYC2a* in plants with dysfunction of COI1 (COI1-RI/MYC2-OE). (**C**) Soluble sugar content of the trichome secretion droplets in the plants of COI1-RI and COI1-RI/MYC2-OE. (**D**) Comparison of cembratriene-diol contents between the plants of COI1-RI and COI1-RI/MYC2-OE. (**E**) The representative chromatogram profiles of cembratriene-diol determination by the UPLC system for the plants of COI1-RI and COI1-RI/MYC2-OE. The triangle indicates the unidentified compound observed in above results. (**F**) Transcription profiles of the cembranoid synthetic genes *LTP1*, *CBTS* and *P450* in the plant set of the control (Ctrl), COI1-RI and COI1-RI/MYC2-OE. The transcription level of each gene in the control (Ctrl) plants was arbitrarily set as “1”. Values in (**C**,**D**,**F**) are the average of data from three independent lines with triplicate measurements. Asterisks in (**C**,**D**,**F**) indicate significant difference from the plants of COI1-RI. Error bars, mean ± SE.

To investigate the regulation of cembratriene-diol formation by the regulators functioning downstream of the JA-Ile receptor, the plants with dysfunction of COI1 were employed for further assays. The tobacco homologue of *Arabidopsis MYC2*, i.e., bHLH transcription factor *NtMYC2a*, was overexpressed in the tobacco with dysfunction of COI1. Trichome staining with I_2_/KI showed that overexpression of *NtMYC2a* had no obvious effect on the volume of trichome secretion droplets, but turned the secretion droplets to a darker color and increased the soluble sugar content of the trichome secretions, as well (Figure 5A–C). Following cembratriene-diol determination revealed that overexpression of *NtMYC2a* notably enhanced the cembratriene-diol production of plants with dysfunction of COI1 (Figure 5D,E). The transcriptional analyses suggested that overexpression of *NtMYC2* accentuated the transcription levels of *LTP1*, *CBTS* and *P450* genes in the plants with dysfunction of COI1 (Figure 5F). This evidence suggested the involvement of NtMYC2 in regulating COI1-mediated cembratriene-diol formation in tobacco.

### 2.5. Regulatory Roles of NtMYB305 in COI1-Mediated Cembratriene-Diol Formation in Tobacco

**Figure 6:**
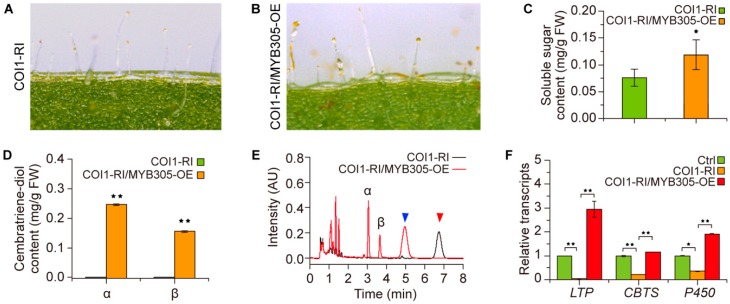
Regulatory roles of NtMYB305 in COI1-mediated cembratriene-diol formation in tobacco. (**A**,**B**) Trichome secretion droplets staining with I_2_/KI for plants with dysfunction of COI1 (COI1-RI) and overexpressing *NtMYB305* in plants with dysfunction of COI1 (COI1-RI/MYB305-OE). (**C**) Polysaccharide content of the trichome secretion droplets in the plants of COI1-RI and COI1-RI/MYB305-OE. (**D**) Comparison of cembratriene-diol contents between the plants of COI1-RI and COI1-RI/MYB305-OE. (**E**) The representative chromatogram profiles of cembratriene-diol determination by the UPLC system for the plants of COI1-RI and COI1-RI/MYB305-OE. The triangles indicate the unidentified compounds observed in the analyzed samples. (**F**) Transcription profiles of the cembranoid synthetic genes *LTP1*, *CBTS* and *P450* in the plant set of Ctrl, COI1-RI and COI1-RI/MYB305-OE. The transcription level of each gene in the control (Ctrl) plants was arbitrarily set as “1”. Values in (**C**,**D**,**F**) are the average of data from three independent lines with triplicate measurements. Asterisks in (**C**,**D**,**F**) indicate significant difference to the plants of COI1-RI. Error bars, mean ± SE.

Furthermore, the tobacco homologue of *Arabidopsis* MYB24, i.e., MYB transcription factor NtMYB305, was also overexpressed in the tobacco with dysfunction of COI1. Staining with I_2_/KI showed that overexpression of *NtMYB305* resulted in a pronounced increase in the volume of trichome secretion droplets of plants with dysfunction of COI1, and subsequent analysis also revealed an increase in the soluble sugar content of the trichome secretions (Figure 6A–C); whereas no obvious color change in the trichome secretion droplets was observed (Figure 6A,B). Further determination showed that the contents of cembratriene-diol of the trichome secretions was obviously increased by overexpression of *NtMYB305* in tobacco with dysfunction of COI1 (Figure 6D,E). The transcription levels of *LTP1*, *CBTS* and *P450* genes were also accentuated by overexpression of *NtMYB305* (Figure 6F). This evidence suggested the involvement of NtMYB305 in regulating COI1-mediated cembratriene-diol formation in tobacco. Interestingly, an unidentified compound observed in the above results was abolished by overexpressing *NtMYB305* in the plant with dysfunction of COI1, but a novel unidentified compound appeared with the retention time shifted to 5 min from 6.8 min.

## 3. Discussion

The therapeutic activities of cembranoids in cancer and inflammation treatment, as well as their fascinating architectures are attractive to pharmaceutical developers [1,2]. Currently, the majority of cembranoids from plants were identified from conifers and tobacco [1], and tobacco is the most feasible plant that could be applied to dissect the molecular mechanism of cembranoids’ synthesis in plants. Thus, the studies of JA-induced cembranoid formation in this study are of great importance for the utilization of plant-derived cembranoids. 

Many studies have proven that JAs display important roles in regulating the secondary metabolism in plants [18,19,20,21] and also act as regulators in controlling the initiation and secondary metabolism of glandular trichomes, which are the factories producing cembranoids [4,17,22,23,40,41]. In this study, treatment of tobacco seedlings with MeJA could not only increase the secretion level of glandular trichome, but also promote the formation of cembratriene-diols, which evidenced the regulatory function of JA in cembranoid formation. Previous studies identified several genes involved in cembranoid synthesis, *CBTS* (cembratrienol synthase), *P450* (cytochrome P450 hydroxylase) and *LTP1* (lipid transfer protein) [1,8,9], and the qRT-PCR analyses carried out in this study demonstrated that MeJA treatment could significantly enhance the transcription levels of *CBTS*, *P450* and *NtLTP1* genes in tobacco. This finding implied that JA functions as positive regulator of cembratriene-diol formation via upregulating the cembranoid synthetic genes. 

COI1 is a critical component of the SCF^COI1^ receptor complex of the JA-signaling pathway, which displays diverse roles in the regulation of JA-mediated plant growth and development, defense response and secondary metabolism [20,26,27,28]. In this study, we analyzed the roles of tobacco COI1 in regulating cembranoid synthesis using previously developed transgenic plants [25], and the results showed that dysfunction of COI1 decreased the cembratriene-diol content of tobacco leaves to an undetectable level. Subsequent research found that the transcription levels of *CBTS*, *P450* and *NtLTP1* genes were significantly decreased in plants with dysfunction of COI1 compared to that in the control plants. These results evidenced the involvement of COI1 in regulating the formation of cembratriene-diols and the transcription of cembranoid synthetic genes including *CBTS*, *P450* and *NtLTP1*. Since the COI1-mediated JA-signaling network was well established [28,42,43,44,45], the involvement of the JA perception complex in cembranoid synthesis will provide important clues to uncover the regulatory mechanism underlying cembranoid formation.

Previous studies established that COI1 and JAZ proteins are the key components of the JA-Ile receptor complex and play pivotal roles in governing the JA responses in plants [20,26,27,28]. The WD40-bHLH-MYB complex acts as a critical modulator in controlling the downstream JA responses, including secondary metabolism and transcriptional regulations [10,14,15,36,37,38,39], in which the bHLH (such as MYC2) and MYB transcription factors are the direct targets of JAZ proteins and exert important functions in mediating JA responses [27,29,30,46]. Thus, it is of great importance to study the functions of JA-responsive bHLH and MYB transcription factors in regulating cembranoid synthesis, which may provide direct evidence of the molecular regulation of cembranoid synthesis. To analyze the roles of NtMYC2a and NtMYB305 in regulating cembranoid synthesis, *NtMYC2a* and *NtMYB305* were overexpressed in the tobacco with dysfunction of COI1, respectively. The studies showed that *MYC2*-overexpression could notably enhance the cembratriene-diol production of plants with dysfunction of COI1, but with no obvious effects on the trichome secretion. *MYB305*-overexpression not only increased the cembratriene-diol production, but also increased the quantity of the trichome secretion of plants with dysfunction of COI1. Moreover, the transcriptional assays revealed that overexpression of *MYC2* or *MYB305* could increase the transcriptional levels of cembranoid synthetic genes including *CBTS*, *P450* and *NtLTP1* in the plants with dysfunction of COI1. Thus, these findings demonstrated that MYC2 and MYB305 function as positive regulators of cembratriene-diols synthesis, but these two kinds of regulators worked in distinctive patterns. As mentioned above, bHLH transcription factor MYC2 and MYB transcription factor MYB305, the tobacco homologues of *Arabidopsis* MYB21 and MYB24 [47], are the direct targets of JAZ proteins and function downstream of COI1 to regulate secondary metabolism in plants [31,34,46,47,48,49,50,51]. The enhancement of cembratriene-diol production, as well as the upregulation of cembranoid synthetic genes in plants with dysfunction of COI1 by overexpression of *MYC2* or *MYB305* evidenced that MYC2 and MYB305 both function downstream of COI1 in regulating cembratriene-diol synthesis in tobacco, which is consistent with their function patterns in regulating other JA responses [10,14,15,27,29,30,36,37,38,39,46].

## 4. Materials and Methods

### 4.1. Plant Materials

Tobacco cultivar *Nicotiana tabacum* L. cv. TN90 was used in this study. The *NtCOI1*-silenced tobacco plants and the empty-vector-transformed control plants were developed in our previous study [25].

The *NtMYC2a*- and *NtMYB305*-overexpressing plants were generated using *Agrobacterium*-mediated transformation with *Agrobacterium tumefaciens* LBA4404 carrying the target binary vectors, which were constructed as following. The binary vectors to express *NtMYC2a* and *NtMYB305* were constructed by inserting the cDNAs of *NtMYC2a* (amplified with primers 5′-ATGACTGATTACAGCTTACCC-3′ and 5′-GCGTGTTTCAGCAACTCTGGA-3′) and *NtMYB305* (amplified with primers: 5′-ATGGATAAAAAACCATGCAAC-3′ and 5′-ATCGCCGTTAAGCAATTGCAT-3′) into a 2X35S promoter carrying binary vector pBIN19-attR-HA, which was modified from pBIN19-attR-YFP [52], by Gateway^®^ cloning, as the manufacture’s introduction (Invitrogen, Carlsbad, USA) to get the vectors pBIN19-NtMYC2a-HA and pBIN19-NtMYB305-HA, respectively. 

The hybrid plant of *NtCOI1*-silenced and *NtMYC2a*-overexpression was generated by crossing of the *NtCOI1*-silenced plants with pollens from *NtMYC2a*-overexpressing plants. The hybrid of *NtCOI1*-silenced and *NtMYB305*-overexpression was generated in a similar manner.

### 4.2. Plant Cultivation and Phytohormone Treatment

All tobacco plants were grown at 23 °C in a greenhouse with a photoperiod of 14 h light/10 h dark. For phytohormone treatment with jasmonate (JA), the 4-week-old seedlings of wild type TN90 were sprayed daily with MeJA aqueous solution (100 μM) for seven days, while the seedlings of control treatment were sprayed with distilled water simultaneously. The leaf samples were collected at indicated time points for further analyses.

### 4.3. Visualization of Trichome Secretion Droplets by Iodine Staining

To visualize the trichome secretion droplets, tobacco leaves were stained by I_2_/KI solution (1% (*w*/*v*) I_2_ in 3% (*w*/*v*) KI), as previously described [25]. The stained leaves were observed under the stereoscopic microscope and photographed. A darker color indicates higher soluble sugar content of the tested samples.

### 4.4. Soluble Sugar Content Determination

The soluble sugar content of the trichome secretion was determined using the method for total soluble sugar measurement with anthrone reagent as previously described [53]. Briefly, the soluble sugar of glandular trichome secretion was extracted with anhydrous alcohol by washing the leaf sample, and then, 1/4 V (volume) distilled water was added into the extraction for a dilution. Following that, 0.5 mL of extraction were mixed with 2.5 mL of ice-cold anthrone solution (0.2% (*w*/*v*) anthrone in 72% (*v*/*v*) H_2_SO_4_) and incubated in the water-bath of 100 °C for exactly 11 min. Then, the reactions were transferred onto ice immediately to stop the reaction. The absorbances of the reactions were measured at 630 nm, and the soluble sugar content of each sample was calculated as previously described [53].

### 4.5. Measurement of Cembratriene-Diols

The cembratriene-diol content of tobacco glandular trichome secretion was determined using the UPLC (ultra performance liquid chromatography) system. In brief, the cembratriene-diol of glandular trichome secretion was extracted by washing tobacco leaves with ethyl acetate and dried in the nitrogen flow. Then, the extraction was dissolved in the solvent of 80% acetonitrile in water (*v*/*v*) and filtrated through filters with a membrane with a 0.22-μm pore size for injection (10 µL). The chromatography assay was performed on an ACQUITY UPLC system (Waters, Milford, CT, USA) under the following optimized conditions: BEH C18 column (1.7 µm, 2.1 mm × 100 mm) with the column temperature of 35 °C, a mobile phase of gradient acetonitrile (Table 1) at the flow rate of 0.3 mL min^−1^ and a UV detector for the detection of cembratriene-diols at 200 nm. Cembratriene-diol purified from tobacco leaves with preparative LC (liquid chromatography) system and verified by the MS spectrum was used as the standard. Standard curves were made for quantification by serial dilutions of the cembratriene-diol stock solutions (1 mg/mL).

### 4.6. Quantitative RT-PCR (qRT-PCR)

Samples for total RNA extraction were collected from fully-expanded leaves of the indicated plants. Total RNAs were extracted using TRIzol reagent (Invitrogen) according to the manufacturer’s instructions. First-strand cDNAs were synthesized using the PrimeScript™ II 1st Strand cDNA Synthesis Kit (TaKaRa, Dalian, China) and used as templates for qRT-PCR. Reactions were performed using an ABI 7500 real-time PCR system with GoTaq^®^ qPCR Master Mix (Promega, Madison, USA).

Primers for quantification of target genes in qRT-PCR assays are as follows: 5′-AGCAAGATTGCATGTTTCGTG-3′ and 5′-CCAGCAAATAAGGGACGCAA-3′ for *LTP1* (GenBank Accession: AB625593); 5′-TCAGACTGCATCCTCCACTACC-3′ and 5′-CTCCTTCCGCTACCAAAGGG-3′ for *P450* (GenBank Accession: AF166332.1); 5′-ATGAGAGTGCACGACGAGGA-3’ and 5′-CCTTGCTCCCACCCTTGGTA-3′ for *CBSTs* (GenBank Accessions: HM241151, HM241152, HM241153); 5′-CCACACAGGTGTGATGGTTG-3′ and 5′-GTGGCTAACACCATCACCAG-3′ for *Actin* (GenBank Accession: X63603). The *Actin* gene was used as an internal control. The relative transcripts were obtained by calibrating the threshold cycles of the genes of interest with that of *Actin* using the equation 2^(–ΔΔCT)^ [54], where C_T_ is the cycle number of the threshold point at which fluorescence is detectable.

### 4.7. Statistical Analysis

Data in the figures are presented as the mean ± SE. Student’s *t*-test was carried out for the statistical analyses of cembratriene-diol and soluble sugar contents and for the qRT-PCR data. Differences in the tests were considered statistically significant at a *p*-value < 0.05 (*) or <0.005 (**).

## 5. Conclusions

This study has demonstrated a critical role of JA, as well as COI1, the receptor protein of the bioactive derivative of JA (i.e., JA-Ile), in governing cembratriene-diol synthesis in tobacco. Findings of this study suggested that JA-signaling pathways may control the cembratriene-diol formation in tobacco via manipulating not only the formation of trichome secretion, but also its composition, which involves the function of downstream bHLH and MYB transcription factors. However, *NtMYC2a* and *NtMYB305* exhibit distinctive roles in regulating the cembratriene-diol formation and trichome secretion in tobacco. 

## Figures and Tables

**Table 1:** Gradient acetonitrile concentrations.

Time (min)	Acetonitrile Concentration (*v*/*v*)
0	50%
1	60%
2	70%
3	75%
4	90%
5	100%
6	90%
7	80%
8	60%
9	50%
